# A Web-Based Mobile App With a Smartwatch to Support Social Engagement in Persons With Memory Loss: Pilot Randomized Controlled Trial

**DOI:** 10.2196/13378

**Published:** 2019-06-18

**Authors:** Hayley R McCarron, Rachel Zmora, Joseph E Gaugler

**Affiliations:** 1 Division of Epidemiology and Community Health School of Public Health University of Minnesota Minneapolis, MN United States; 2 Division of Health Policy and Management, School of Public Health, University of Minnesota Minneapolis, MN United States

**Keywords:** Alzheimer disease, dementia, social support, quality of life, well-being, technology, social engagement, facial recognition, smartwatch

## Abstract

**Background:**

It is estimated that the number of individuals living with dementia worldwide will increase from 50 million in 2017 to 152 million by 2050. Assistive technology has been recognized as a promising tool to improve the lives of persons living with memory loss and their caregivers. The use of assistive technology in dementia care is expanding, although it is most often intended to manage care and promote safety. There is a lack of assistive technology designed to aid persons with memory loss in participating in meaningful activities. The Social Support Aid (SSA) is a mobile phone-based app that employs facial recognition software. It was designed to assist persons with memory loss remember the names and relationships of the people they interact with to promote social engagement.

**Objective:**

This study uses a pilot randomized controlled trial (RCT) design to evaluate the SSA. The objectives were to ascertain (1) the feasibility and utility of the SSA, (2) whether the outcomes of SSA use suggest potential benefits for persons living with memory loss and their care partners, and (3) how study design components could inform subsequent RCTs.

**Methods:**

Persons with memory loss were randomized to the SSA (n=20) or the usual care control group (n=28). Quantitative data were collected at three timepoints (baseline, 3 months, and 6 months). Participants in the intervention group participated in qualitative interviews following completion of their 6-month survey.

**Results:**

Participant eligibility, willingness to be randomized, and retention were not barriers to conducting a full-scale RCT; however, recruitment strategies should be addressed before doing so. Feasibility and utility scores indicated that participants felt neutral about the technology. Use of the SSA was not significantly associated with changes in quality of social interactions or quality of life measures over the 6 months of follow-up (*P*>.05). The qualitative analysis revealed three themes that described how and why the SSA worked or not: (1) outcomes, (2) reasons why it was or was not useful, and (3) recommendations.

**Conclusions:**

There is a need to develop effective assistive technology that improves the quality of life of persons with memory loss. Assistive technology that allows persons living with memory loss to maintain some level of autonomy should be a priority for future research. This study suggests reasons why the SSA facial recognition software did not appear to improve the quality of social interaction and quality of life of people with memory loss. Results also provide recommendations for future assistive technology development and evaluation.

**Trial Registration:**

ClinicalTrials.gov NCT03645694; https://clinicaltrials.gov/ct2/show/NCT03645694 (Archived by WebCite at http://www.webcitation.org/78dcVZIqq)

## Introduction

Globally, the number of older persons aged 60 years and older is projected to more than double by 2050 and triple by 2100, increasing from 962 million in 2017 to 2.1 billion in 2050 and 3.1 billion in 2100 [1]. As the number of older adults increases throughout the world, so too will the prevalence of dementia [2,3]. The percentage of persons living with Alzheimer disease increases dramatically with age: 3% of people ages 65 to 74 years, 17% of people ages 75 to 85 years, and 32% of people ages 85 years or older have Alzheimer disease. In the absence of a medical breakthrough to prevent, slow, or cure Alzheimer disease and other dementias, it is estimated that the number of individuals worldwide living with the disease will increase from 50 million in 2017 to 152 million by 2050 [2,4,5].

As there is no cure for Alzheimer disease and other dementias, efforts to develop interventions and resources that improve the lives of persons living with dementia and their caregivers are a public health priority [6]. Assistive technology has been recognized as a promising avenue for such improvements and holds potential as a tool to promote the autonomy of persons with dementia by enabling their daily activities [7-11]. Assistive technology in dementia care can be defined as an item, piece of equipment, product or system driven by electronics that is used to help individuals or their caregivers manage the consequences of dementia [12]. Assistive technology has been shown to improve independence, behavior symptoms, and quality of life as well as reduce caregiver stress in randomized controlled trials (RCTs). Further, studies suggest persons with dementia generally have positive feelings about using assistive technology to promote their independence [6,12,13].

Although the use of assistive technology in dementia care is rapidly growing, such devices are often intended to assist caregivers rather than the person with dementia [12-15]. Most assistive technology in the context of dementia care is used for delivering assessments, assisting with activities of daily living (ADLs), safety, or in managing care. Few evaluations of assistive technology designed to enhance social well-being exist. This is particularly problematic given that one of the most pressing challenges for persons living with dementia and their caregivers is finding meaningful activities to engage in [8,12,14,16]. Further, it is essential that persons living with dementia have some level of autonomy for as long as possible when participating in meaningful activities, such as socializing, to maintain good quality of life [10]. Assistive technology may provide an opportunity for persons living with dementia to participate in meaningful and engaging activities, but the benefits of assistive technology in these domains remains unclear [10,12,14].

This study is a pilot RCT (NCT03645694) evaluating the potential of an assistive technology device, the Social Support Aid (SSA). The principal objective of this pilot randomized controlled evaluation was to ascertain (1) how participants perceived the feasibility and utility of the SSA, (2) whether the outcomes of SSA use suggest potential benefits for persons living with memory loss and their care partners, and (3) how the various study design components could inform subsequent larger-scale RCTs. This study fills a gap in the literature by evaluating the potential for an assistive technology device designed to aid persons with memory loss engage in meaningful social interactions.

## Methods

### Design

A pilot RCT design was used. A pilot RCT is generally employed to determine whether the elements required for conducting a successful, full-scale RCT are present. Specifically, a pilot RCT determines whether screening eligibility procedures operate effectively, recruitment targets are met, randomization is carried out appropriately and selection bias is mitigated, whether the intervention is carried out as intended, and if the intervention is sufficiently intense to result in the anticipated benefits [17]. An important objective of a pilot RCT is also to highlight challenges when conducting the intervention.

An underpowered RCT is not a pilot RCT [17]. It is important to note that this study was not designed as a pilot RCT a priori. However, the extent of qualitative and feasibility/utility data that were collected over the 6-month evaluation of the SSA allowed us to address many of the core objectives that are often posited in pilot RCTs. For this reason, we chose to label this project as a “pilot” RCT.

### The Social Support Aid Technology

**Figure 1:**
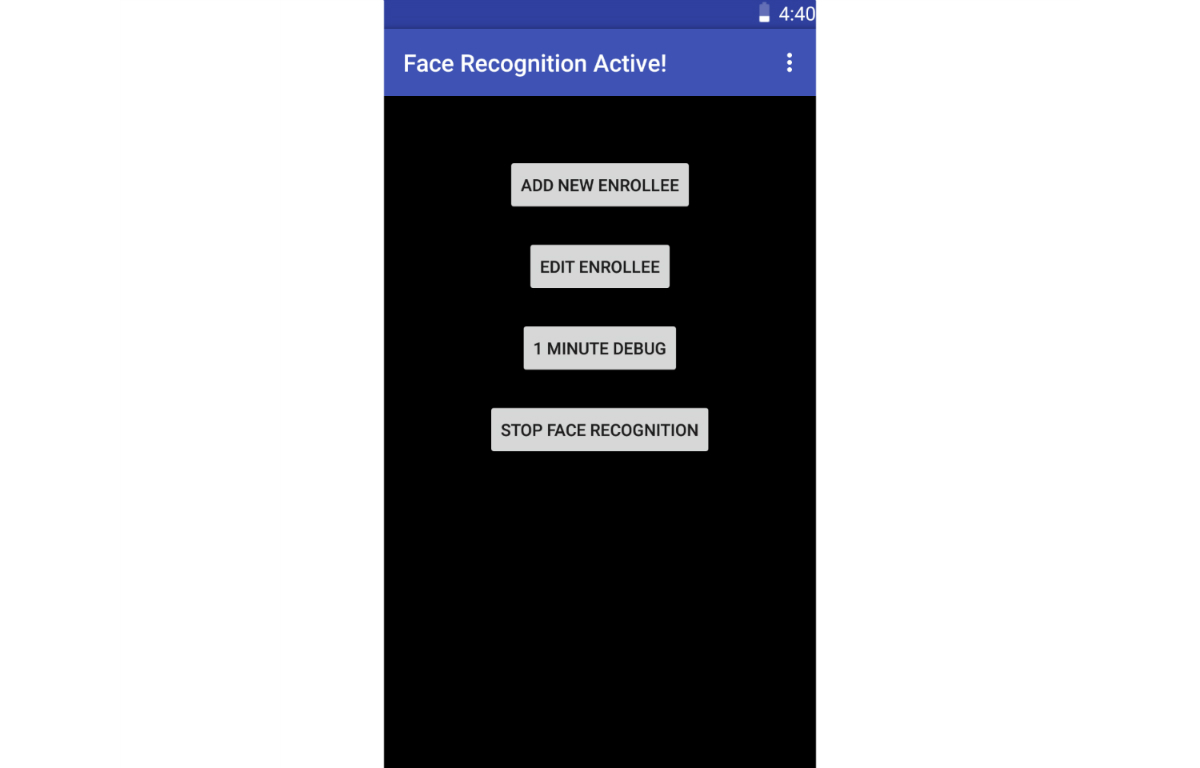
The Social Support Aid app home screen.

**Figure 2:**
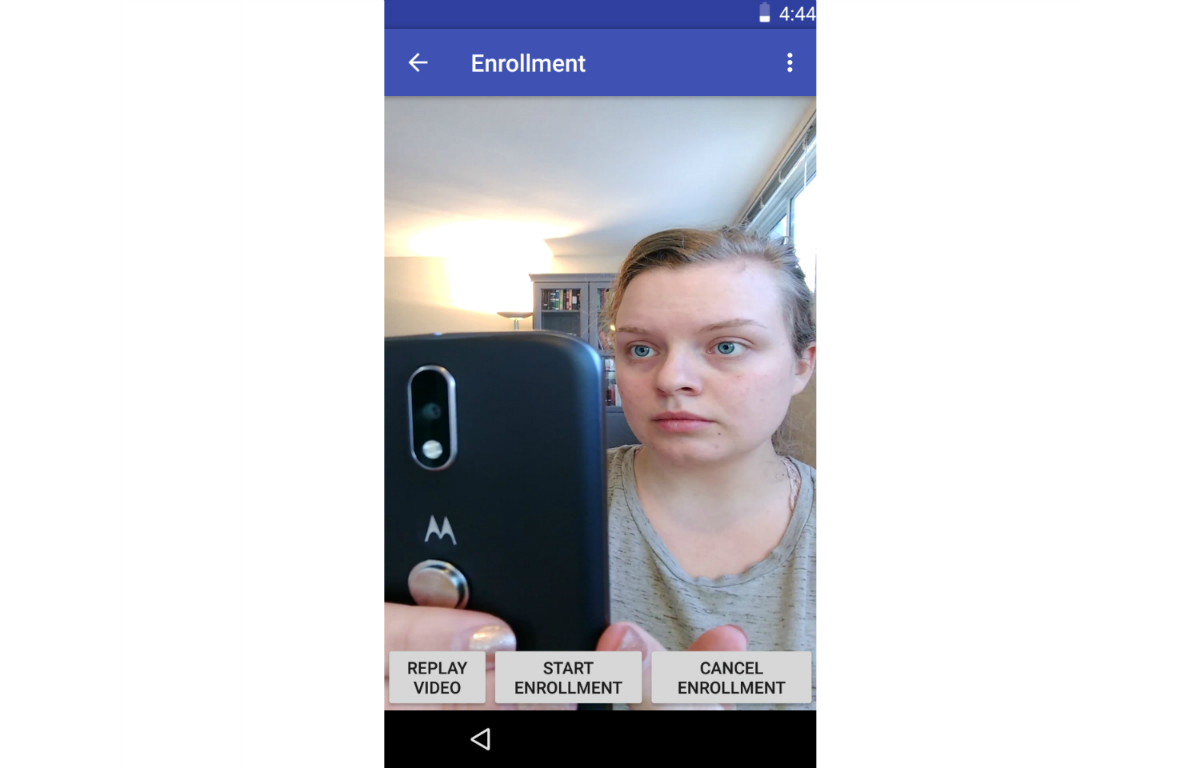
The Social Support Aid app enrollment instruction video.

**Figure 3:**
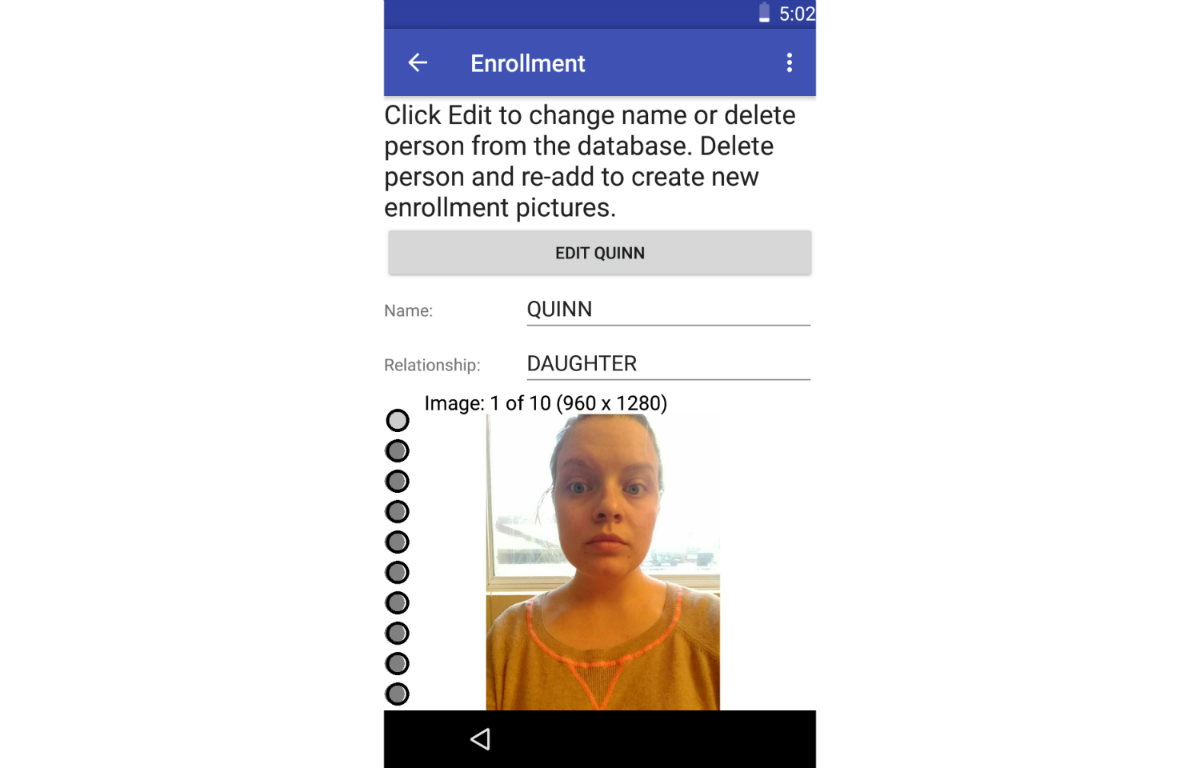
The Social Support Aid app enrollment screen.

**Figure 4:**
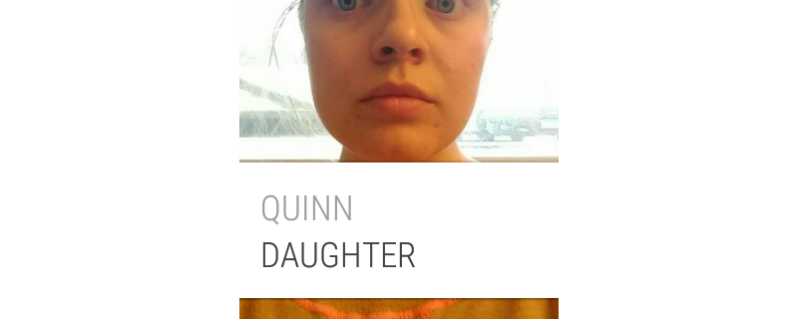
The Social Support Aid watch face after the app has recognized the face.

The SSA is a mobile phone-based app that employs facial recognition software. The SSA technology was developed by Advanced Medical Electronics, a research and development company specializing in medical devices. The SSA was designed to assist persons with memory loss remember the names and relationships of the people they interact with to promote social engagement. The technology consists of a mobile phone equipped with a facial recognition software app and a smartwatch. Up to 1000 individuals can be “enrolled” in the facial recognition app database. Enrollment includes typing an individual’s name and relationship to the person with memory loss into the app and taking pictures of the individual’s face from multiple angles. Once enrolled and in view of the mobile phone’s camera, the SSA app recognizes the individual’s face and alerts the smartwatch. The watch then vibrates and displays the individual’s image and text with their name and relationship to the person with memory loss. For pictures of the device, see Figure 1-4.

During phase 1 testing of the SSA, 14 participants (seven dyads of persons with dementia or mild cognitive impairment and their caregivers) provided feedback on the SSA. Participants were given a demonstration of the SSA and were trained to use it. Participants were then walked through the SSA again and were asked a series of guided questions to elicit their opinions of the SSA. Qualitative data collected from the initial testing indicated that participants thought the technology would be useful in social situations and that they understood how to operate the SSA. Given the initial positive results, a more rigorous review was warranted. Results presented in this study are from the second phase of testing.

### Recruitment

Individuals with dementia, memory loss, or memory concerns, as well as their caregivers, were recruited from the University of Minnesota Caregiver Registry (a registry of caregivers who gave permission to be contacted about opportunities to participate in research), the Minnesota State Fair, and through statewide newspaper advertisements from February to October 2017. Participants included caregivers and persons with memory loss. The following inclusion criteria were applied: (1) ability to fill out a survey in English or Spanish; (2) 21 years or older; (3) diagnosis of dementia or mild cognitive impairment, or has a self-identified memory concern (or a caregiver of such an individual); and (4) person with memory loss has sufficient cognitive capacity to provide verbal informed consent (measured by score of 20 or higher on St Louis University Mental Status examination).

Caregivers provided Health Insurance Portability and Accountability Act (HIPAA) authorization and written informed consent, and persons with memory loss provided assent. In instances where there was no caregiver available, persons with memory loss provided HIPAA authorization and written informed consent to participate. Participants were given US $100 following their completion of the study. The University of Minnesota’s Institutional Review Board approved this study. This study was registered with ClinicalTrials.gov following clarification of the trial status and design (NCT03645694).

### Data Collection

Data were collected at three time points: baseline, 3 months, and 6 months. Caregivers completed all surveys on behalf of the person with memory loss. Participants were asked for their opinion of their relative with memory loss (eg “How often *does your relative* feel confident?”). At baseline, surveys for participants caring for a person with memory loss measured ADLs, memory impairment, memory and problem behaviors, social interaction, and quality of life as well as demographic questions asking about themselves and the person with memory loss. Participants with memory loss who did not have a caregiver completed surveys on their own behalf. They received a slightly different version of the survey with questions being asked in reference to themselves (eg, “How often *do you* feel confident?”). Their baseline survey measured ADLs, memory impairment, social interaction, and quality of life as well as demographic questions about themselves. Surveys administered at 3 and 6 months were identical to the baseline surveys except that they did not include demographic questions. At 3 and 6 months, participants in the intervention group completed an additional feasibility and utility checklist. Participants were given the option to complete an online or paper version of the surveys.

Following completion of the baseline survey, participants were randomly assigned to either receive the technology or to continue with usual care. Participants were randomized at a ratio of 1:1 using a random number generator. Neither the participants nor researchers were blinded to randomization group. Research assistants met with participants in the intervention group in-person to provide the mobile phone and smartwatch and demonstrate how to use the SSA technology. Participants were given the technology to use at their discretion, and there was no requirement for how many times they had to use the SSA. Throughout the study, research assistants and the SSA developer provided technical support and answered questions regarding the technology. Participants in the control group were given the technology free of charge after completing the study.

### Analysis

#### Recruitment, Randomization, and Retention

Chi-square and *t* tests were used to determine if participant demographics in the intervention and control groups were significantly different (*P*<.05). Chi-square and *t* tests were also used to compare participants who were lost to follow-up with those who were not.

#### Feasibility and Utility

Participants in the intervention group were asked to complete an additional survey at 3 and 6 months to assess their perceptions of feasibility and utility. This checklist included 15 Likert scale items asking participants to rate their level of agreement with statements such as “the technology works well,” “SSA was easy to use,” and “my relative felt lost using SSA” (ɑ=.89).

#### Assessment of Intervention Effect

Descriptive statistics were calculated for measures of quality of social interaction and quality of life. Social interaction quality was measured by asking participants to rate their satisfaction with the following types of communication: visits, phone calls, mail correspondence, and computer correspondence. Quality of life was measured using the Pleasant Events Schedule-Alzheimer’s Disease (PES-AD; frequency ɑ=.84; enjoyment ɑ=.76) and Dementia Quality of Life (DQoL; ɑ=.92). The PES-AD asks with what frequency and level of enjoyment the person with memory loss experiences a list of pleasant activities (eg, being outside, listening to music, laughing). The DQoL asks participants to use a Likert scale to rate how often the person with memory loss feels a certain way (eg, satisfied, cheerful, angry, worried).

We imputed missing data using a Markov chain Monte Carlo method to conduct a five-fold multiple imputation. Analyses were conducted as intention to treat. Change scores were calculated to determine differences between outcomes at baseline and 6 months. To determine whether changes in satisfaction and quality of life in the intervention group were significantly different than changes in the control group, *t* tests were used. Statistical significance was assessed using two-tailed tests with a significance level of *P*=.05.

#### Qualitative Analysis

Following completion of the 6-month survey, participants in the intervention group were asked to participate in a semistructured interview; 13 individuals agreed to participate. The interviews took place over the phone and lasted between 10 and 30 minutes each. Interviews were transcribed by a professional service and organized into NVivo. Qualitative data were coded using Braun and Clarke’s [18] six steps of thematic analysis. HM first read through all transcripts and then generated initial themes. HM and JG discussed and compiled codes into an initial coding framework. Next, HM coded all material and revised the coding framework as needed. The qualitative analysis was guided by the research question: How and why did the SSA work or not work for caregivers and persons with memory loss?

## Results

### Recruitment, Randomization, and Retention

**Figure 5:**
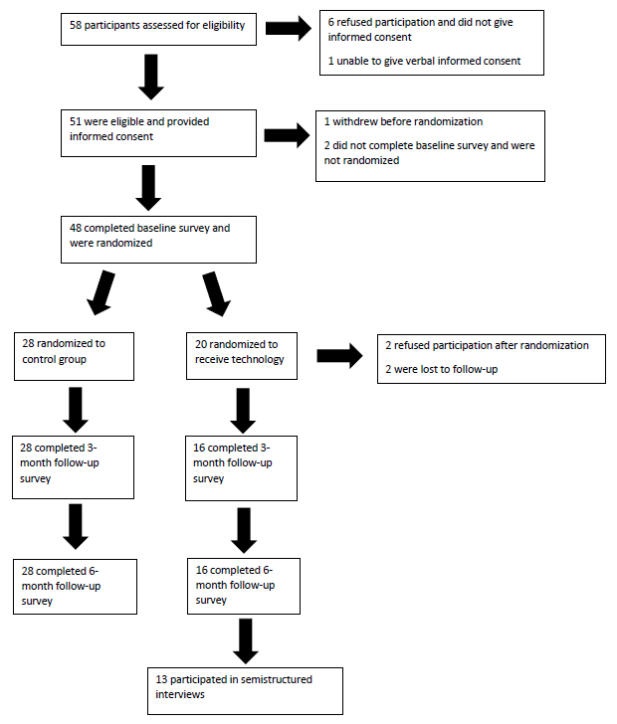
Participant flow diagram.

Recruitment was a challenge despite the use of newspaper advertisements and community outreach. A total of 58 potential participants were assessed for eligibility; of these, all but one met the inclusion criteria. Six of the 58 potential participants were unwilling to provide informed consent and were not included in the study. None of the participants expressed unwillingness to be randomized. There were no statistically significant differences in participant demographics between the intervention and control groups (Tables 1 and 2), suggesting successful randomization. Of the 48 participants that were randomized, 44 finished the study (92% retention rate). Two participants with memory loss refused participation after undergoing randomization to the intervention group. Two participants who were caregivers were lost to follow-up, both in the intervention group (Figure 5). Participants lost to follow-up were significantly different with regards to randomization group and income, with participants in the intervention group and caregivers with an income of US $10,000 to US $14,999 and US $80,000 and over being more likely to be lost to follow-up.

Thirty-five participants were caregivers and 13 were persons with memory loss who had no caregiver available. Persons with memory loss were an average age of 74.90 (SD 6.98) years. The majority of persons with memory loss were non-Hispanic white (40/47, 85%), married or living with their partner (32/47, 68%), and had been diagnosed with dementia (29/48, 60%; see Table 1). Caregivers were an average age of 67.83 (SD 10.08) years. The majority were female (25/35, 71%), non-Hispanic white (30/34, 88%), and were caring for their spouse or partner (28/35, 80%; Table 2).

### Feasibility and Utility

Mean feasibility and utility scores were calculated at 3- and 6-month follow-ups. The mean score at 3 months was 3.11 (SD 0.57) and at 6 months was 3.10 (SD 0.63), which suggested moderate feasibility and utility (items were scored one through five, with lower scores indicating less favorable perceptions of SSA’s utility and higher scores more favorable). The item receiving the highest score was “the information provided on how to use SSA was clear to me” (3 months: mean 4.07, SD 0.62; 6 months: mean 4.06, SD 0.68). The item receiving the lowest score was “after using SSA, I feel like my relative is more at ease in social situations” (3 months: mean 2.71, SD 0.73; 6 months: mean 2.5, SD 0.97).

### Assessment of Intervention Effect

A total of 48 participants were included in the analytic sample. The use of SSA was not associated with significant changes in PES-AD, DQoL, or measures of social interaction satisfaction (Table 3).

### Qualitative Results

The qualitative analysis resulted in three themes that described how and why the SSA worked or did not: (1) outcomes, (2) reasons why it was or was not useful, and (3) recommendations. Participant names were replaced with pseudonyms when reporting results.

#### Outcomes

This theme describes the impact using the SSA had on caregivers and persons with memory loss. The majority of participants did not think their use of the SSA had any effect, although some mentioned positive and negative aspects of using the SSA.

##### Positive Outcomes

Most participants who thought their use of the SSA had an influence perceived the SSA in a positive fashion. Some participants stated that the SSA gave their relative confidence and independence, such as Marsha (caregiver, age 83), who said:

I wasn’t always providing backup and that gave him more confidence...So he didn’t have to rely on me giving cues or asking me any questions because he was able to use it and found an answer himself. I think that’s important.

For others, such as Kelley (caregiver, age 72), using the SSA was beneficial in that it provided a topic of conversation:

One of the really neat things about it is those people who we had successfully enrolled in it, they just got such a kick out of it when the phone would recognize them. That was just a delight to them and it was a good conversation opener. It was something that really enhanced our conversations with people.

For some participants the technology was a novelty they enjoyed “tinkering around with” and demonstrating for friends and family.

##### Negative Outcomes

Although the majority of participants felt the SSA had a positive impact or no impact at all, some participants felt that the SSA resulted in negative outcomes. For example, Marge (caregiver, age 67) said that the SSA was an additional distraction, hindering her husband as he attempted to have conversations. Doris (caregiver, age 72) said that her husband’s anxiety “went through the roof” while using the technology. The technology, she said, was too overwhelming and caused him to become agitated. Others reported that using the SSA was a source of frustration and in one case became a point of tension between the caregiver and person with memory loss. Rebekah (caregiver, age 69) explained:

I think it was frustrating and then it got that way for me, too, because I couldn’t keep explaining it and explaining it and demonstrating. Because then it would just get to be a fight, arguing about what it was doing. He just could not quite comprehend [the SSA].

Other caregivers said that the SSA was an additional burden, contributing to an already long list of caregiving duties. For them, the technology was “just one more thing” they had to keep track of.

**Table 1:** Persons with memory loss demographics.^a^

Demographic		Total (N=48)	Intervention (n=20)	Control (n=28)	*P* value
Age (years), mean (SD)		74.90 (6.98)	74.15 (5.22)	75.43 (8.06)	.54^b^
Number of living children, mean (SD)		2.77 (1.94)	2.53 (1.58)	2.96 (2.19)	.47^b^
**Gender, n (%)**					.73
	Female	25 (52)	11 (55)	14 (50)	
	Male	23 (48)	9 (45)	14 (50)	
**Ethnicity, n (%)**					.99
	Non-Hispanic	40 (85)	17 (85)	23 (85)	
	Hispanic	7 (15)	3 (15)	4 (15)	
**Race, n (%)**					.37
	White, non-Hispanic	36 (84)	16 (84)	20 (83)	
	White, Hispanic	2 (5)	2 (11)	0	
	Asian	1 (2)	0	1 (4)	
	≥2 races	3 (7)	1 (5)	2 (8)	
**Marital status, n (%)**					.53
	Married/living with partner	32 (68)	13 (68)	19 (68)	
	Divorced	3 (6)	1 (5)	2 (7)	
	Widowed	8 (17)	2 (11)	6 (21)	
	Separated	3 (6)	2 (11)	1 (4)	
	Never married	1 (2)	1 (5)	0	
**Education, n (%)**					.88
	Less than high school degree	6 (13)	2 (10)	4 (14)	
	High school degree	5 (10)	2 (10)	3 (11)	
	Some college	5 (10)	1 (5)	4 (14)	
	Associate’s degree	4 (8)	2 (10)	2 (7)	
	Bachelor’s degree	9 (19)	5 (25)	4 (14)	
	Graduate degree	18 (38)	8 (40)	10 (36)	
**Annual household income, n (%)**					.33
	<$25,000	11 (25)	6 (32)	5 (20)	
	$25,000-$29,000	4 (9)	0	4 (16)	
	$30,000-$39,000	4 (9)	1 (5)	3 (12)	
	$40,000-$59,000	7 (16)	2 (11)	5 (20)	
	$60,000-$79,000	7 (16)	4 (21)	3 (12)	
	>$79,000	11 (25)	6 (32)	5 (20)	
Lives with caregiver, n (%)		31 (65)	14 (70)	17 (61)	.46
Diagnosed with dementia, n (%)		29 (60)	13 (65)	16 (57)	.58

^a^From nonimputed dataset.

^b^*P* values were computed with *t* test assuming equal variance; otherwise, chi-square test was used.

**Table 2:** Caregiver demographics.^a^

Demographic		Total (N=35)	Intervention (n=15)	Control (n=20)	*P* value
Age (years), mean (SD)		67.83 (10.08)	67.47 (13.33)	68.10 (7.14)	.86^b^
Number of living children, mean (SD)		2.39 (1.69)	2.57 (1.87)	2.26 (1.59)	.61^b^
**Gender, n (%)**					.83
	Female	25 (71)	11 (73)	14 (70)	
	Male	10 (29)	4 (27)	6 (30)	
**Ethnicity, n (%)**					
	Non-Hispanic	34 (100)	15 (100)	19 (100)	
	Hispanic	0	0	0	
**Race, n (%)**					.64
	White, non-Hispanic	30 (88)	14 (93)	16 (84)	
	White, Hispanic	1 (3)	0	1 (5)	
	Asian	1 (3)	0	1 (5)	
	≥2 races	2 (6)	1 (7)	1 (5)	
**Marital status, n (%)**					.16
	Married/living with partner	30 (88)	14 (93)	16 (84)	
	Divorced	3 (9)	0	3 (16)	
	Never married	1 (3)	1 (7)	0	
**Education, n (%)**					.35
	Less than high school degree	2 (6)	1 (7)	1 (5)	
	High school degree	4 (11)	0	4 (20)	
	Some college	4 (11)	3 (20)	1 (5)	
	Associate’s degree	2 (6)	1 (7)	1 (5)	
	Bachelor’s degree	7 (20)	4 (27)	3 (15)	
	Graduate degree	16 (46)	6 (40)	10 (50)	
**Annual household income, n (%)**					.27
	<$25,000	4 (13)	2 (14)	2 (11)	
	$25,000-$29,000	2 (6)	0	2 (11)	
	$30,000-$39,000	2 (6)	1 (7)	1 (6)	
	$40,000-$59,000	4 (13)	0	4 (22)	
	$60,000-$79,000	8 (25)	4 (29)	4 (22)	
	>$79,000	12 (38)	7 (50)	5 (28)	
**Work status, n (%)**					.05
	Working full or part-time	9 (26)	5 (33)	4 (20)	
	Retired	24 (69)	10 (67)	14 (70)	
**Relationship to PWML^c^, n (%)**					.57
	Spouse or partner	28 (80)	13 (87)	15 (75)	
	Child	6 (17)	2 (13)	4 (20)	

^a^From nonimputed dataset.

^b^*P* values were computed with *t* test assuming equal variance; otherwise, chi-square test was used.

^c^PWML: person with memory loss.

**Table 3:** Primary outcomes for persons with memory loss at baseline, 3 months, and 6 months.^a^

Outcome measure	Baseline, mean (SD)		3 months, mean (SD)		6 months, mean (SD)		*P* value
	Intervention (n=20)	Control (n=28)	Intervention (n=16)	Control (n=28)	Intervention (n=16)	Control (n=28)	
PES-AD^b^ (frequency)^c,d^	2.28 (0.29)	2.38 (0.31)	2.19 (0.49)	2.23 (0.33)	2.3 (0.32)	2.24 (0.35)	.92
PES-AD (enjoyment)^c,e^	2.5 (0.26)	2.48 (0.29)	2.43 (0.47)	2.38 (0.34)	2.39 (0.32)	2.34 (0.33)	.88
DQoL^f,g^	3.47 (0.67)	3.58 (0.61)	3.4 (0.63)	3.29 (0.73)	3.38 (0.67)	3.38 (0.57)	.63
Satisfaction with quality of visits^h^	1.84 (0.96)	1.85 (1.13)	1.73 (0.80)	1.85 (0.99)	2.4 (1.19)	1.89 (1.09)	.18
Satisfaction with quality of phone calls^h^	2.05 (1.00)	2.24 (1.13)	2.2 (1.15)	2.44 (1.04)	2.53 (1.3)	2.52 (1.12)	.36
Satisfaction with quality of mail correspondence^h^	2.79 (1.25)	2.62 (0.87)	2.77 (1.30)	3.09 (0.73)	3 (0.88)	2.87 (1.22)	.57
Satisfaction with quality of computer correspondence^h^	2.5 (1.25)	2.18 (1.05)	2.31 (0.95)	2.36 (0.95)	2.72 (1.14)	2.56 (0.96)	.95

^a^For mean (SD), means were calculated from nonimputed dataset; *P* values were calculated from imputed dataset.

^b^PES-AD: Pleasant Events Schedule-Alzheimer’s Disease.

^c^Excluded by error from baseline survey for participants with no caregivers.

^d^1=not at all, 2=1-6 times in the last week, 3=7 or more times in the last week.

^e^1=not at all, 2=somewhat, 3=a great deal.

^f^DQoL: Dementia Quality of Life.

^g^1=never, 2=seldom, 3=sometimes, 4=often, 5=very often.

^h^1=very satisfied, 2=somewhat satisfied, 3=feel neutral, 4=somewhat dissatisfied, 5=very dissatisfied.

#### Reasons Why it Was or Was Not Useful

The majority of participants interviewed did not find the technology useful. Caregivers and persons with memory loss offered a variety of reasons why the SSA was not useful to them. These reasons could be divided into the following subthemes: (1) complexity of the SSA, (2) enrollment process, (3) impracticality, (4) stigma, and (5) functionality of the SSA.

##### Complexity of the Social Support Aid

Several participants felt that the SSA was too complicated and difficult for someone with memory loss to use. Many said that because of memory loss, they had difficulty using technology and learning new things. For example, Kent (person with memory loss, age 73) said:

My ability to use technology, it turns out, is much more diminished than I kind of expected it was. I just had trouble giving people instructions well enough to effectively get them enrolled in the system. I was not a very good guide.

Often, the diminished ability to use technology was compounded by a general discomfort with technology. Doris (caregiver, age 72) explained:

But most [people with memory loss] may not have had very much experience at all with technology and have never had a cell phone, still have their landlines. And so, introducing something that’s so foreign to them, and that they’re intimidated by, at least initially presents an additional challenge.

The concept of the technology posed a problem for some. Doris went on to explain how difficult the concept of the SSA was for her husband, Nathan (age 77), saying:

And in a way it assumes that the person [with memory loss] can make the connection between the name that’s on the watch and the person that’s looking at you...And so just seeing one little row of print on the watch, assuming they remember that that’s where it is, it didn’t connect with Nathan at all. I mean he was like “ok, so now what do I do?”...You know, conceptually it was hard for me to help Nathan understand what was going on, how the two pieces of technology interacted.

Others would forget what the phone and watch were there for, resulting in confusion and agitation.

##### Enrollment Process

The enrollment process was frequently mentioned as a reason why the SSA was not useful. Enrollment consisted of entering an individual’s picture, name, and relationship to the person with memory loss in the SSA facial recognition database. Many said the enrollment process was time-consuming and cumbersome. Kelley (caregiver, 72) described the enrollment process, saying:

When it didn’t work well in capturing their photos, that made it get cumbersome...When they faced the camera at their face and they turned it one way and turned it the other way and all that, if that had accepted their photos it would not have gotten cumbersome. When it started to get cumbersome is when you had to do it and do it again and do it again. There’s only so many times that I felt comfortable trying to ask one person to do that.

Others, such as Doris, felt uncomfortable asking people to enroll in the first place:

The concept of asking people that you want to have in the system to spend a few minutes, you know, getting into the system through that facial recognition process was really awkward...And so there was hesitation on our part who we would ask because it seemed like we were being a little bit intrusive to them.

For these reasons, many said they only felt comfortable asking individuals they knew well to enroll. Consequently, the individuals they enrolled were often people that the person with memory loss did not have trouble remembering. Kelley explained:

If it had been a little bit easier getting them enrolled, then I think it would have been more useful...We were reluctant to reach out to anybody who wasn’t pretty close to us, to get them to put up with that process. There were a number of them who gave it a good try and just never made it [into the database]. We were not able to get them enrolled...The only people we had enter themselves into it were people that we were already reasonably close to and that we really weren’t having any problem remembering. If the circle were a little wider and if we had been able to get some people who were a little more distant from us enrolled, I could see there where it would really help with social interactions.

Others said that the process was not conducive to enrolling others with memory loss or young grandchildren who had a hard time sitting still and following instructions.

##### Impractical

Some participants felt that the SSA was not practical for use in their everyday lives. For example, Marge (caregiver, age 67) said:

It’s like I’ve become this helicopter wife making sure I’m right there...We didn’t use it in a situation where it did anything for me. Like I said, I still had to be right there...I don’t leave him and most of the other caregivers don’t generally leave their significant other either.

For many, their social interactions were not conducive to using the SSA. For example, Marge mentioned that the adult day service and a community chorus group for people with dementia were the only social settings that the SSA could be useful to her husband with memory loss. In both settings, name tags were already worn, limiting the usefulness of the SSA. Others mentioned that it was not practical to use during everyday interactions such as going to the movies, shopping, or going to the gym.

##### Stigmatizing

A few caregivers were concerned that the SSA was too conspicuous. For example, Maria (caregiver, age 34) said,

Well, it didn’t help because [my mother] wouldn’t wear it...She felt having that big phone around her neck just drew a lot of attention to her, which she does not like.

For such participants, the technology was stigmatizing.

##### Functionality of the Social Support Aid

The functionality of the technology includes how well the SSA worked, the physical appearance of the technology, and characteristics of the phone and watch. Several participants reported that the SSA only worked in certain lighting. Some had trouble getting it to work outdoors and in dimly lit settings. Others reported that the SSA only worked when the camera was at particular angles. Marge (caregiver, age 67) mentioned that the software could not distinguish her son from her son-in-law, both of whom were bald and had beards but otherwise had little physical resemblance. Rebekah (caregiver, age 69) thought the SSA took too long to recognize a face. She said that by the time the SSA recognized the face, her husband had already asked her who the person was. Several participants thought the phone was too heavy to have hanging around the neck. Many thought the phone was uncomfortable and not practical for everyday activities. Doris explained:

[My husband goes] to a senior exercise facility. And that’s the most likely place where he’s going to see more than just family. But [the phone] kind of bounces around...He didn’t like that thing on his chest. It was just really awkward...Cause it’s not very secure in that position. It doesn’t stay down. If you stand up it just-or bend over it drops forward, right?...I think it’s kind of dangerous to have it flopping around.

Feedback on the watch was mixed. Some felt it was too bulky, whereas others thought it fit nicely and was esthetically pleasing. Similarly, some felt the watch face was too small to read the text, whereas others thought it was sufficiently large.

#### Recommendations

Although most participants did not find the technology useful in its current state, most felt it had the potential to be beneficial. Many offered recommendations for how the SSA technology could be improved to maximize its usefulness for persons with memory loss and their caregivers. A number of participants recommended improving the enrollment process by allowing users to upload photos of the enrollee’s face instead of taking their picture. Several participants suggested replacing the phone and watch with something less obtrusive and conspicuous. Participants suggested replacing the watch with an earpiece. Arnie (caregiver, 75) recommended:

I have a Bluetooth interface between my hearing aids. Being able to, for instance, have some way of recognizing a face the way this system is designed, and to be able to speak—rather than look at my watch—to be able to hear the name of the person in my ears without even anything more than that would be extremely helpful. Even to someone who has no hearing aids. But being able to put something as inconspicuous [as an] earphone, to be able to connect wirelessly to a system that would recognize a face and put a name to it would be extremely helpful.

Many also felt the phone was too obtrusive. Instead of the phone, they recommended a lapel pin, brooch, pendant, or necklace with a camera.

## Discussion

### Principal Findings

Results indicate that issues of participant eligibility, willingness to be randomized, and retention are not major barriers to conducting a full-scale RCT to evaluate the SSA. However, it is noteworthy that all participants who withdrew from the study or who were lost to follow-up were in the intervention group. There were no significant differences between baseline demographic measures of the groups, suggesting that randomization was successful despite the small sample size.

Feasibility and utility scores for both 3- and 6-month time points were 3.11 and 3.10, respectively, indicating participants felt neutral about the SSA. Our findings also suggest that the SSA may not have significant effects. Due to the small sample size, these results should be interpreted with caution and are subject to further investigation in a larger sample. The absence of empirical intervention effects is supported by the qualitative analysis, which revealed that the majority of participants did not find the SSA useful. Anecdotally, many participants mentioned they were not using the SSA, and a number of participants in the intervention group have contacted the study staff wishing to return the technology since the study ended. The qualitative analysis provides insight into why the SSA had few significant effects and provides recommendations for improving the technology. The majority of the participants interviewed did not feel their use of the SSA had any impact on the person with memory loss’s social interactions or quality of life. A few did note positive outcomes such as increased confidence and independence. Conversely, others mentioned negative outcomes such as increased frustration, agitation, tension between the caregiver and person with memory loss, and caregiver burden.

The qualitative analysis indicates five primary reasons explaining why the SSA was not useful to participants: complexity, the enrollment process, impracticality, stigma, and functionality. Concerns about the complexity, enrollment process, and functionality of the SSA are consistent with similar evaluations of assistive technology reporting usability and technical reliability as barriers to use among persons with memory loss. Assistive technology that requires wearing any form of equipment has been found to be stigmatizing in other studies (particularly for persons with memory loss); however, increased attention to esthetics may reduce the stigmatization of wearable assistive technology [12,19,20].

### Future Research

Based on the findings of this pilot study, a full-scale RCT should invest significant time and resources in recruitment. In this study, recruitment was a challenge despite the use of newspaper advertisements and community outreach. Future assistive technology research in this population may consider partnering with community-based organizations to recruit participants. Subsequent research on assistive technology should also measure time spent using the technology and participants’ level of comfort with technology.

Although most participants reported having limited use for the SSA, almost all were enthusiastic about its potential benefit to persons with memory loss. Several offered suggestions for modifications to make it more useful. Before a full-scale RCT is conducted on the SSA, modifications recommended by participants should be addressed. Specifically, the process of enrolling users in the SSA database should be made less cumbersome and the SSA equipment should be replaced with less obtrusive and conspicuous options.

Findings from this pilot study highlight the importance of user-centered design and testing for future development of assistive technology in dementia and memory loss care. It is imperative that future assistive technology development goes beyond understanding theoretical causes and implications for cognitive impairment to understand what the person with memory loss wants from the technology [8]. Persons with memory loss and their care partners should be involved early in the process of assistive technology development [12]. As is evident in this pilot RCT, their insights should be incorporated in any future adaptation, full-scale evaluation, and dissemination of the SSA or similar technologies.

### Strengths and Limitations

Assistive technology can give persons living with memory loss the ability to participate in meaningful and engaging activities; however, scientific evaluation of such assistive technology use remains limited [10,12,14]. This study fills a gap in the literature by evaluating the potential for an assistive technology device designed to improve the social interactions of individuals with memory loss. A strength of this study is the inclusion of both caregivers and persons with memory loss, incorporating the perspectives of all intended users. The study also included Spanish-speaking participants, allowing for a more ethnically diverse sample.

This study also has a number of limitations. As noted previously, this study was not considered a pilot RCT a priori, and it could be considered an underpowered RCT due to the challenges reported here (eg, small sample size, recruitment/enrollment issues, little evidence that the SSA exerts meaningful effects on key outcomes). Another limitation is that potentially important feasibility/utility outcomes such as time spent using the SSA and prior technology use were not collected because the study was not designated as a pilot a priori. However, the robust qualitative data available allowed us to reach a key conclusion more aligned with a pilot RCT design: that the SSA may require significant modification before it could proceed to a full-scale RCT and as an intervention that could exert both statistically and clinically significant benefits for persons living with memory loss and their care partners.

Another potential limitation is the inclusion of individuals with mild cognitive impairment and subjective memory loss in addition to those with a diagnosis of dementia. Although individuals with subjective memory loss may experience the intervention differently than individuals with dementia, we felt it was important to include individuals without a formal diagnosis. According to the Alzheimer’s Association, a substantial proportion of those who would meet the diagnostic criteria for dementia are not given a diagnosis by a physician. Further, fewer than half of Medicare beneficiaries in the United States who have a diagnosis of dementia in their health records report being told of the diagnosis. As such, a large number of individuals living with dementia and their caregivers may not know they have dementia (at least in the United States) [2].

The accidental exclusion of the PES-AD from the baseline survey of participants with memory loss is another limitation. This error resulted in no baseline measure of PES-AD for the 13 participants with memory loss; however, all other measures of quality of life and social interaction were not impacted by this omission. Another limitation is that neither participants nor researchers were blinded; however, in a study such as this it would not have been feasible to render the intervention blind. Additionally, qualitative data were collected via telephone interview, which may not allow for the exploration of the user’s experience of the SSA as an in-person interview would. Finally, the study has limited racial and ethnic diversity despite the translation of materials into Spanish and the inclusion of a Spanish-speaking research assistant.

### Conclusions

Many effective assistive technologies have been developed to improve the management of care and quality of life for caregivers of persons with memory loss [21-23]; however, there is a need to develop effective assistive technology that improves the quality of life of persons with memory loss. This study indicates that randomization procedures were sound but that retention and recruitment procedures should be addressed before scaling up to an RCT. The assessment of intervention effects suggests that the SSA may not exert significant effects on quality of life and social interactions. Feasibility and utility data reveal that participants had generally neutral feelings toward the SSA. Qualitative findings suggest reasons why the facial recognition software did not improve outcomes and provide recommendations for future assistive technology development and evaluation.

One of the most prominent challenges for caregivers and persons living with memory loss is finding meaningful activities to engage in. Assistive technology that allows persons living with memory loss to maintain some level of autonomy when socializing or participating in desired activities harbors potential to maintain quality of life and remains a priority for future experimental research efforts.

## Abbreviations

DQoL: Dementia Quality of Life
HIPAA: Health Insurance Portability and Accountability Act
PES-AD: Pleasant Events Schedule-Alzheimer’s Disease
RCT: randomized controlled trial
SSA: Social Support Aid

## Appendix

Multimedia Appendix 1 CONSORT‐EHEALTH checklist (V 1.6.1).
